# RETRACTION: The effect of preoperative mechanical bowel preparation in paediatric bowel surgery on postoperative wound related complications: A meta‐analysis

**DOI:** 10.1111/iwj.70132

**Published:** 2024-11-12

## Abstract

**RETRACTION**: ChenM. , LinJ. , MiaoD. , YangX. , FengM. , LiuM. , XuL. , LinQ. , “The effect of preoperative mechanical bowel preparation in paediatric bowel surgery on postoperative wound related complications: A meta‐analysis,” International Wound Journal 21, no. 4 (2024): e14884, 10.1111/iwj.14884.38654483 PMC11040098

The above article, published online on 23 April 2024, in Wiley Online Library (http://onlinelibrary.wiley.com/), has been retracted by agreement between the journal Editor in Chief, Professor Keith Harding; and John Wiley & Sons, Ltd. Following an investigation by the publisher, the parties have concluded that this article was accepted solely on the basis of a compromised peer review process. Therefore, the article must be retracted. The authors disagree with the retraction.

**RETRACTION**: M. Chen , J. Lin , D. Miao , X. Yang , M. Feng , M. Liu , L. Xu , Q. Lin , “The effect of preoperative mechanical bowel preparation in paediatric bowel surgery on postoperative wound related complications: A meta‐analysis,” International Wound Journal 21, no. 4 (2024): e14884, 10.1111/iwj.14884.38654483 PMC11040098

The above article, published online on 23 April 2024, in Wiley Online Library (http://onlinelibrary.wiley.com/), has been retracted by agreement between the journal Editor in Chief, Professor Keith Harding; and John Wiley & Sons, Ltd. Following an investigation by the publisher, the parties have concluded that this article was accepted solely on the basis of a compromised peer review process. Therefore, the article must be retracted. The authors disagree with the retraction.

